# Analysis of the Prediction of Motivational Climate in Handball Players’ Fear of Failure

**DOI:** 10.3390/ijerph16030344

**Published:** 2019-01-26

**Authors:** Manuel Gómez-López, Victoria Ruiz-Sánchez, Antonio Granero-Gallegos

**Affiliations:** 1 Department of Physical Activity and Sport, Faculty of Sport Sciences, University of Murcia, Santiago de la Ribera, 30720 Murcia, Spain; mgomezlop@um.es; 2 Human Performance and Sports Science Research Group (E0B5-05), Faculty of Sport Sciences, University of Murcia, Santiago de la Ribera, 30720 Murcia, Spain; victoria.ruiz2@um.es; 3 Department of Education, Faculty of Education Sciences, University of Almeria, 04120 Almeria, Spain; 4 Health and Public Administration Research Center, University of Almeria, 04120 Almeria, Spain

**Keywords:** shame, coach, peer, mastery, avoidance behaviours

## Abstract

Sports can at times become a negative socializing agent for athletes. The objectives here were to analyse the relationship between motivational climates (involving coaches and peers) and fear of failure in players according to gender and sports experience, and also to control for the extent to which these motivational climates predict the different aversive causes of fear of failure. To this aim, a non-experimental, descriptive, and sectional design was used in which participants answered the Perceived Motivational Climate in Sport Questionnaire, the Peer Motivational Climate in Youth Sports Questionnaire, and the Performance Failure Appraisal Inventory. The sample included 479 handball players aged 16–17 years old (mean = 16.60; standard deviation = 0.50) who were playing in Spanish regional youth category handball teams. The results revealed that the task-involving training climate predominates in both genders over the ego-involving training climate, particularly in girls and in less experienced players. The peer ego-involving climate also predominates with respect to the peer task-involving climate in both genders, but this is particularly so for boys and in more experienced players. Furthermore, except for fear of feeling shame, which was predicted by the peer ego-involving climate, all the aversive causes of fear of failure are predicted mainly by the coach climate. The immediate environment was proved to be determinant in avoidance behaviours and fear of failure.

## 1. Introduction

Throughout players’ training processes, organized competitive sports have social influences, and these influences affect training and the intention to continue playing [1]. In this context of skills and achievements in which athletes try to reach a goal and in which proving competence and ability is important, Roberts and Treasure [2] claim that physical and psychological well-being depends on the contexts where sports practice is carried out, which are closely related to coaches, parents, peers, and mass media.

At early age stages, results show that players’ parents and family have the strongest influence on children’s involvement in sports practice and on the creation of sports habits [3]. An increase of peer influence is observed during the first stage of adolescence given that they provide support and social recognition, and this role is shared with the figure of the coach as the main agent of influence through the motivational climate perceived in the sports environment [4,5]. With increasing age, peer influence, especially involving players of the same gender, gradually increases throughout adolescence and even exceeds that of parents and coaches [6,7].

More specifically, in the case of a team sport, such as handball, and at the height of adolescence, peers and coaches are the main social agents in a team [8] and they play a central role when it comes to achieving sporting excellence [9,10,11,12]; they are responsible for promoting a certain type of climate during training and thus affect the way players face the tasks proposed [13].

According to Achievement Goal Theory [14,15], the motivational climate refers to the set of signals generated by family, friends, colleagues, and coaches, etc., which is perceived by athletes in their environment, and through which the key factors for success and failure are identified. The relationship between different motivational climates is not clear. Authors such as Biddle et al. [16] and Papaioannou [17] argue that motivational climates are independent, while others, such as Kavussannu and Roberts [18], argue that climates are negatively related.

Thus, according to Achievement Goal Theory [15] either an ego-involving or a task-involving motivational climate will be created according to how the athletes perceive the context [19,20]. In this regard, if victory and showing good skills and performance are the most important aspects for the people in the players’ environment, they create an ego-involving climate, while if they see effort, personal improvement, and skill development as central aspects, they will create a task-involving climate [21]. One of the triggers of avoidance behaviours and fear of failure is the motivational climate created during the adolescent socialization process. An athlete’s ability to manage and control this situation depends on many factors, and poor management can lead to errors or mistakes [7].

While the sports environment is one of the most suitable contexts for achievement as athletes are motivated to succeed by showing their competence and skills [15,22], different authors claim that it is also a medium that can reflect athletes’ incompetence in front of their peers [23]. This is related to the fact that many athletes experience fear of failure in highly competitive environments (e.g., national or regional tournaments), when their performance is judged by referees, coaches, parents, peers, and spectators on the basis of performance and success criteria [24].

Thus, sports practice can generate a sensation of fear of failure and feelings of shame, which in turn create insecurity, anxiety, and stress in athletes as well as avoidance behaviours in relation to what people may say or think about them, especially during the course of a game, which results in lower performance [7,25,26]. Therefore, the fear of making mistakes has become a variable of ever-greater interest in sports studies.

Fear is a subjective emotion, a state of mind or a feeling that has an environmental background and behavioural consequences. Fear of failure appears when a player allows other people to be responsible for controlling their behaviour by looking for their approval and/or fearing disapproval [13]. Thus, failure in itself would not carry negative connotations if it were not for outside judgment and the aversive consequences for athletes’ self-esteem. These consequences of failure can be perceived in a different way by athletes and thus they will affect them in diverse ways. Those who perceive failure consequences as aversive will see failure as a threat and will be afraid of it [24]. As a result of this fear, athletes feel less competent and try to avoid achievement situations [15]. 

Fear of failure triggers an assessment of the negative consequences (anxiety or depression) on the player’s well-being with respect to those potential emotions that players may feel after not performing a task correctly in front of their peers, coaches, or teachers [24,27,28]. Some elite athletes may be reluctant to reveal their fears of failure as they associate fear of failure with weakness of character and lack of success and confidence [24]. Fear of failure therefore affects the well-being, interpersonal behaviour, and sporting performance of athletes [29].

Regarding the assessment of fear of failure, the Performance Failure Appraisal Inventory (PFAI) was opted for [27,28]. The PFAI is the most widely used multidimensional scale today and its purpose is to understand the perception of the aversive consequences of fear of failure in the competitive context [30]. Based on the original scale of 41 items [27], other revised and improved versions of 25 and 5 items subsequently emerged [28]. The 25 items of the scale group the aversive consequences into five dimensions, which will be explained in greater detail later in the Instruments section. As for the reduced scale of 5 items, it is made up of the most representative items of each of the five dimensions. 

It should also be noted that the PFAI, originally designed for North American sports, has been validated in different languages and countries, including the British [31], Spanish [7], Chinese [32], Portuguese [33,34,35], and Danish [36] contexts, and recently for Turkish [37] and Jordanian [38] conditions, as well as in education with Spanish secondary school students in physical education classes [39,40].

Based on the abovementioned information and on the extensive review carried out, it is necessary to highlight the importance of this present study since, to date, there are no published studies in the sports field in Spain attempting to analyse the three variables studied altogether here, and furthermore, this is a preliminary study to guide future research in which the main objective would be to carry out individualized psychological interventions with coaches to improve satisfaction with the sports practice of their players.

Thus, this study had the following objectives: to analyse the relationship between motivational climates (coaches and peers) and fear of failure in players according to gender and sports experience, and also to control for the extent to which these motivational climates predict the different aversive causes of fear of failure.

The following hypotheses (H) were formulated on the basis of a review of the different research projects:

- (H1) The majority of players perceive greater coach and peer task-involving motivational climates and that the main aversive causes of fear of failure will be shame and devaluing one’s self-esteem.

- (H2) The perception of both coach and peer task-involving climates will be negatively related to all aversive causes of fear of failure, while the perception of both coach and peer ego-involving climates will be positively related to all aversive causes of fear of failure.

- (H3) The perception of both coach and peer ego-involving climates will be greater in boys and in players with greater sporting experience, while the perception of coach and peer task climates will be greater in girls and in those players with less federated-level sporting experience. 

- (H4) The aversive causes of fear of failure will be stronger in boys than in girls, with shame and devaluing one’s self-esteem being most commonly expressed caused by women, while uncertain future, losing the interest of important others, and upsetting important others are emotions mostly expressed by men.

- (H5) Coach and peer ego-involving climates will positively predict the different aversive causes of fear of failure.

## 2. Materials and Methods

### 2.1. Participants

A non-probabilistic convenience sampling was used on this research. The sample included a total of 479 youth category handball players (250 boys and 229 girls) selected to compete in the Spanish Regional Championships. These players are rated “high-performance athletes” by the Spanish Sports Council according to Royal Decree 971/2007, of July 13, on high-level and high-performance athletes. The age range was 16–17 (40.1% and 59.9%, respectively) (mean, M = 16.60; standard deviation, SD = 0.50) years old. With regard to the years of experience variable, 85.4% stated that they had more than five years of experience at the federated handball player level; 97.9% trained three or more times per week; and 90% carried out more of three hours of training per week. Apart from the total sample (479), six questionnaires were discarded because they were incomplete.

### 2.2. Measurement Instruments

The Perceived Motivational Climate in Sport Questionnaire-2 (PMCSQ-2) [19,41]. The Spanish version of PMCSQ-2. [42,43] includes 29 items grouped in two dimensions measuring the ego-involving (competitive) climate (14 items, e.g., “On this team, the coach gives most of his or her attention to the stars”), and the task-involving (mastery) climate (15 items, e.g., “On this team, the coach emphasizes always trying to do your best”). Each item was headed with the phrase “In my training group or team…”. Answers were collected on a Likert-type scale ranging from “strongly disagree” (1) to “strongly agree” (5). The internal consistency analysis was satisfactory for the two subscales: mastery (α = 0.87), and competitiveness (α = 0.85).

The Peer Motivational Climate in Youth Sport Questionnaire (PeerMCYSQ) [44]. The Spanish version of the PeerMCYSQ [4] includes 21 items grouped in two second-order dimensions: task-involving climate (12 items, e.g., “They help one another to improve”) and ego-involving climate (9 items, e.g., “They motivate each other to win competitions”). Each item was headed with the phrase “In this team, most of the players...” The answers were collected on a Likert-type scale ranging from “totally disagree” (1) to “totally agree” (7). The internal consistency analysis was satisfactory for the two subscales: task (α = 0.89) and ego (α = 0.70).

The Performance Failure Appraisal Inventory (PFAI) [28]. The Spanish long version of the PFAI [7] includes 25 items grouped in five dimensions: fear of experiencing shame and embarrassment (e.g., “When I am failing, it is embarrassing if others are there to see it”); fear of devaluing one’s self-estimate” (e.g., “When I am failing, it is often because I am not smart enough to perform successfully”); fear of having an uncertain future (e.g., “When I am failing, I believe that my plans for the future will change”); fear of important others losing interest (e.g., “When I am not succeeding, some people are not interested in me anymore”); and fear of upsetting important others (e.g., “When I am failing, important others are disappointed”). All items were headed by the phrase “In my sports practice…” The answers were collected on a Likert-type scale ranging from “do not believe at all” (1) to “believe 100% of the time” (5). Here the internal consistency analysis was satisfactory for the different sub-scales; fear of experiencing shame and embarrassment, α = 0.85; fear of devaluing one’s self-esteem α = 0.70; fear of having an uncertain future, α = 0.83; fear of important others losing interest, α = 0.84; fear of upsetting important others, α = 0.81.

### 2.3. Procedure

The study was carried out during the Spanish Regional Championships. The Royal Spanish Handball Federation, the Handball Federation of Andalusia and the coaches of the different regional selections all granted permission prior to our data collecting after reading a letter explaining the objectives of the study and the way it would be carried out. A sample of the instrument was provided for them all. 

Another letter of presentation was also provided to parents and players. Athletes who were minors were also asked to provide informed written consent from themselves and from their parents, guardians or legal representatives. Once the informed consent had been obtained, the questionnaire was administered.

Data collection was carried out at the hotels where the teams were staying during players’ time off, in agreement with the coaches and in the presence of one of the researchers. Each participant had 20-30 minutes to complete the questionnaire and they were all briefed on the objectives of the study and on their rights as participants, on the voluntary nature of the study and on the confidentiality of answers and data management. They were also informed that there were no correct or incorrect answers and were asked to give true and honest replies. Following data verification, the following variables were recorded: gender, playing position, and years of experience in handball. The research was approved by the Bioethics Committee of the Murcia University (ID: 1494/2017).

### 2.4. Data Analysis

Different analyses were conducted to calculate the descriptive statistics, variance homogeneity, sub-scale correlation, internal consistency of each sub-scale (Cronbach’s alpha), variance analysis (MANOVA), regression, and skewness and kurtosis indices, the latter being in general close to zero and <2, respectively, as recommended by Bollen and Long [45], which indicates a standard univariate normal distribution. All the calculations were carried out with SPSS Statistics V23.0 (SPSS Inc., Chicago, IL, USA).

## 3. Results

### 3.1. Descriptive and Correlation Analysis

Table 1 shows the descriptive values for each of the variables in this study. The results show that the majority of players claim to perceive the coach climate to be more task-oriented than ego-oriented. By contrast, when it comes to the peer climate, the ego-oriented climate showed higher median values than task-oriented climate. In terms of the perception of aversive causes of fear of failure, the results show that the main cause was fear of feeling shame. 

As to the correlation, in the case of PMCSQ-2 the results reveal a significant positive association between the coach ego climate and peer climate (both ego- and task-involving), as well as all the aversive causes of fear of failure, especially fear of important others losing interest (0.42; *p* < 0.001). With regard to the coach task climate, it has a negative relationship with all the aversive causes of fear of failure and with peer ego climate, whereas it had a significant positive relationship with peer task climate (0.46; *p* < 0.001). In the case of the PFAI, all the aversive causes had a significant positive relationship with each other; here, the relationship between fear of experiencing shame and embarrassment and fear of devaluing one’s self-esteem, as well as between fear of important others losing interest and fear of upsetting important others are worth highlighting. As to the correlation of the PFAI scales and PeerMCYSQ, the highest significant positive correlations were observed between the five dimensions of fear of failure and peer ego-involving climate, the highest being (0.40; *p* < 0.001) in the case of fear of important others losing interest (see Table 1).

### 3.2. Differences According to Players’ Gender and Years of Experience

It was controlled for gender and years of sports experience interaction effects on the variables studied with a MANOVA 2X2 (gender x years of sports experience) in which the gender and years of sports experience were independent variables and the PMCSQ-2, PFAI, and PeerMCYSQ sub-scales were dependent variables. First, variance homogeneity was analysed with Box’s M Test. The result revealed rejection of the null hypothesis (Box’s M = 196.48, *F* = 1.35, *p* = 0.004). Following Tabachnick and Fidell [46], Pillai’s Trace was used instead of Wilks’ lambda to analyse the multivariate significance of principal effects and the interactions. The multivariate contrast did not reveal statistically significant differences in the interaction effects between the two independent variables (gender x years of sports experience) (Pillai’s Trace = 0.03, F (9, 467) = 1.59, *p* = 0.115). However, significant gender-related differences were observed (Pillai’s Trace = 0.14; F (9, 467) = 8.23; *p* < 0.001; partial eta squared = 0.14); though that was not the case in terms of years of sports experience (Pillai’s Trace = 0.03; F (9, 467) = 1.43; *p* = 0.173; partial eta squared = 0.03).

In relation to the gender variable, the inter-subject tests revealed significant differences in the following sub-scales: coach ego climate in PMCSQ-2, fear of important others losing interest in PFAI, and peer ego climate in PeerMCYSQ. Boys showed higher medians than girls in all three factors, though effect size values were low (see Table 2).

In terms of years of sports experience, inter-subject effects revealed statistically significant differences in three PFAI scales, but none in PMCSQ-2 or PeerMCYSQ. Players with more than five years of experience showed higher levels of concern in terms of fear of experiencing shame and embarrassment, fear of devaluing one’s self-esteem, and in fear of having an uncertain future than those with less experience. However, effect size values were low (see Table 2).

### 3.3. Regression Analysis

A stepwise regression analysis was carried out in order to find out the extent to which the coach ego climate and the coach task climate (predicting variables) predict fear of failure behaviours (criteria variables). The tolerance and independence indices of the variables included in the regression equation were assessed. The tolerance index was 0.82 and the variance inflation factor (VIF) was 1.21. These values reject the probability of error derived from potential colinearity [47]. Likewise, the Durbin–Watson test statistic (1.60–1.96) reveals data independence [48].

The linear regression analysis results are shown on Table 3. Overall, the coach ego climate in the PMCSQ-2 and the peer ego climate in the PeerMCYSQ were shown to be the predicting variables of the different variables in the PFAI.

In the case of fear of experiencing shame and embarrassment, the peer ego climate was the main predictor (beta = 0.25; *p* < 0.001), while in the second regression step the coach ego climate also needs to be taken into account as a predictor, as it reaches 10% of the explained variance. In the case fear of devaluing one’s self-esteem, the results showed that the higher the coach ego climate, the higher the probability (beta = 0.27; *p* < 0.001) of experiencing this fear, while in the second step the peer ego climate was added as a predicting variable. In fear of an uncertain future, the coach ego climate is also the most relevant predicting variable (beta = 0.35; *p* < 0.001) with 12% of the explained variance. In terms of fear of important others losing interest, the first regression step explained 18% of the variance, and the higher the coach ego climate, the higher was the probability of experiencing this fear. In the second step it reached 23% of the explained variance and the peer ego climate is added as a predicting variable, while in a third step the coach task climate was a predictor, though in this case a negative one. That is, a coach task climate would decrease the fear of important others losing interest. Finally, in relation to fear of upsetting important others, the coach ego climate was the main predictor (step 1), while in the second step the peer ego climate was added as predictor of this fear, reaching 16% of the explained variance (Table 3).

## 4. Discussion

Competitive sport is by nature a manifest and explicit exhibition of the level of performance of athletes in front of the people that surround them, i.e., spectators, coach and colleagues, the relationship with them being considered important. Horn and Weiss [49] highlight the great influence of peers along with coaches, parents, or teachers. Therefore, the objectives of the study were to analyse the relationship between motivational climates (coaches and peers) and fear of failure in players according to gender and sports experience, and also to control for the extent to which these motivational climates predict the different aversive causes of fear of failure.

The results revealed that the sample surveyed mainly perceived a higher coach task-involving climate in relation to the ego-involving climate, which is in line with previous studies showing a similar trend with team sports samples (e.g., [10,12,50,51,52,53,54,55]).

These are positive results in view of the fact that so far the literature has shown that coaches who create a task-involving climate promote effort, interest in learning, and personal progress through comparison to oneself to value competence level, skill development, and mutual cooperation in their team players [56]. Furthermore, different studies have shown that sports players subject to this type of motivational climate enjoy psychological well-being, increase their enjoyment of sports practice and team performance, and show lower levels of competitive anxiety, as opposed to players training in a competitive or ego-based climate, who experience high anxiety levels and get less satisfaction in sports practice [57].

Another important aspect is that those players who perceive a coach task-involving climate in their training, see defeat as a learning factor and that success is achieved with effort, as opposed to those subject to an ego climate, for whom ability and tricks are the main tools to succeed in sports [20,58]. Furthermore, coaches who promote a task climate see mistakes as part of players’ learning process, a stance that is transmitted to their players who thus feel lower levels of fear of failure, as recently shown by Moreno-Murcia and Conte [7] in a study with basketball players.

In terms of peer climate, the results revealed that the majority of players had a high perception of ego climate, which is in line with previous results in Marques et al. [53] for football players. However, our results contradict those in Torregrosa et al. [55] and Leo et al. [59] for team sports. 

Most of the studies examining the analysis of both motivational climates (coach and peer) in team sports have revealed that the perception of both coach and peer task climate is higher than the perception of an ego climate [55,59]. Furthermore, our results are in line with those in Marques et al. [53], which confirm that regardless of coaches, and even if they have a positive approach to training, the peer motivational climate needs to be taken into account given that, as has been shown, a perception of an ego-involving motivational climate can be created. 

In terms of the aversive causes of fear of failure, shame and devaluing oneself were proved to be the main ones. These results are partially in line with previous studies. Conroy [60] and Sagar et al. [61] claim that fear of feeling shame is at the core of fear of failure followed in the view of these authors by fear of an uncertain future, fear of upsetting important others, fear of important others losing interest, and finally fear of devaluing oneself. In fact, Sagar and Stoeber [62] revealed that the only significant predictor in relation to fear of failure was fear of feeling shame. Gustafsson et al. [63] have recently shown that fear of feeling shame is associated with high levels of psychological stress, which would have an impact on fear of failure.

Thus, and based on all the above, the first hypothesis formulated in the study, which stated that the majority of handball players would claim to mainly perceive both a coach and peer task-involving climate and that the main aversive causes of fear of failure would be shame and devaluing one’s self-esteem, is partially verified.

The second hypothesis was that the perception of both a coach and peer task-involving climate would be negatively related to all the aversive causes of fear of failure, while both coach and peer ego-involving climates would be positively related to all the aversive causes of fear of failure. The results obtained confirmed this hypothesis since the correlational analysis showed that there is effectively a positive association between the ego climate and all the aversive causes of fear of failure, the highest being fear of important others losing interest, and a negative association between coach task climate and peer ego climate along with all the aversive causes of fear of failure. This is in line with the study carried out by Boixadós et al. [64], which suggested that the two coach motivational climates can coexist simultaneously in a given context.

It is worth noting that a review of the literature did not reveal any studies associating the perception of these two motivational climates and fear of failure in the sports context. Likewise, the number of studies in the literature relating any of these two motivational climates to the different aversive causes of fear of failure in athletes is very low [7,32].

In this regard it is necessary to highlight the study carried out by Tsai and Chen [32], who examined the perception of the coach motivational climate and the fear of failure in Chinese adolescent athletes, as well as Moreno-Murcia and Conte [7], who carried out a study with Spanish basketball players in which peer climate and the fear of failure were analysed. The results of the correlational analysis in this study are in line with those found by Tsai and Chen [32], in which a positive relationship was found between the coach ego climate and fear of failure, although they did not find positive or negative significant values between the coach task climate and fear of failure. This also coincides with the results found by Moreno-Murcia and Conte [7], which revealed that the peer task climate positively predicted intrinsic motivation in players, and this in turn negatively and significantly predicted fear of failure.

The third hypothesis, formulated according to the perceived motivational climates, proposed that the perception of both a coach and peer ego climate would be greater in boys and in players with greater sports experience, while the perception of a task climate would be greater in girls and in those players with less federated sports experience. The results show that the coach task climate was prevalent in boys and girls, and that it is higher in girls and in players with fewer years of experience, while the peer ego climate was prevalent in both boys and girls, though it was higher in boys and slightly higher in players with more years of experience. On the basis of these results, this hypothesis is partially confirmed for the coach task climate, but not for the peer task climate and is also confirmed for the peer ego climate, but not for the coach ego climate.

Although, as previously mentioned, no studies have been found that jointly relate both motivational climates (coach and peer) and the fear of failure, other studies have been found that address some of these agents separately in team sports.

In terms of the coach motivational climate and players’ gender, the results were in line with previous studies showing that boys perceive higher coach ego climate than girls, who perceive a more task-involving climate [18,55,65,66,67,68,69,70,71]. These results, which only refer to the coach climate, show that boys still tend to interpret sports as a competitive context while girls have better internalized the cooperative and leisure aspect of sports. However, as mentioned in the introduction, the influence of peers grows at the start of adolescence, sharing prominence with the figure of the coach as the main influence agent due to the perception of the motivational climate in the environment [4,5]. 

Furthermore, Torregrosa et al. [55] showed that in the case of boys, the coach is the crucial agent playing a decisive role while girls see their peers as the main agent. This means that potential interventions should be gendered, with the focus on coach training in the first case and on group intervention in the case of girls. On the other hand, some studies found no significant differences in terms of coach climate and the gender variable [54,72].

As to those studies analysing the perception of peer motivational climate and gender, the results show that girls perceived significantly higher task-involving climates than boys and that boys perceived higher ego-involving climates [55]. However, the results obtained for the different regional handball team players revealed that although boys perceived higher levels of ego climate—in line with the studies mentioned above—there was no coincidence in terms of task climate level, as boys also show higher percentages than girls. These results reveal high levels of competition among players in the same team, especially in boys, when the bar is as high as it is in the context of regional and national teams.

To conclude this analysis on motivational climates and the different variables included in the study, it is necessary to emphasize that after an extensive bibliographic review no research has been found with results related to the perception of motivational climate and level of sports experience.

The fourth hypothesis claimed that the aversive causes of fear of failure would be stronger in boys than in girls, shame and devaluing one’s self-esteem being most commonly expressed by women, while an uncertain future, losing the interest of important others, and upsetting important others are causes mostly expressed by men.

As to fear of failure and gender, the literature reveals that boys have higher levels of fear of failing than girls [21,73,74], which is in line with the results for the handball teams analysed here. However, there are discrepancies in relation to the main aversive causes of fear of failure, which are gendered. The results showed confirm the hypothesis and reflect the fact that sports roles are still perceived differently by boys and girls, even in terms of the perception of fear of failure given that boys still attribute more importance to external factors such as social acceptance and social image, while girls are more focused on internal factors and attribute less importance to the opinion of others and more importance to feeling good about themselves. 

Furthermore, this fourth hypothesis held that the aversive causes of fear of failure would be more common in players with more sporting experience. To conclude this analysis it is worth noting that no studies were found revealing results related to the perception of motivational climate and fear of failing according to players’ sports experience. Different studies showed that athletes who practised physical activity more frequently or had more experience showed less fear of making mistakes than those who spent less time practicing sports [75,76].

The fifth hypothesis was that both coach and peer ego climates would positively predict the different aversive causes of fear of failure. The results found in the present study confirm this hypothesis, and also that except for fear of feeling shame, predicted mainly by peer ego climate, all the others were predicted mainly by coach ego climate. Similar results were found by Moreno-Murcia and Conte [7] with a sample of basketball players.

The results showed that the peer task climate positively predicted intrinsic motivation and this in turn negatively predicted fear of failure. These results are of great importance for the planning of potential intervention programs in regional teams aiming at promoting a task-involving climate, given that at this age and in this sports context the coach has been shown to have more influence than peers on the different aversive causes of fear of failure.

It is worth noting that while this study contributes new information on the relationship between coach and peer motivational climate and fear of failing in players, it also has some limitations. First, it is recognized that the size of the sample analysed, even though it is made up in its entirety of the best youth-category handball players in Spain, limits the generalizability of the results, which should be regarded as preliminary as they need to be replicated at other levels of sports performance and in other categories. Future studies should include an analysis of the influence of players’ parents on the development of fear of failure through the motivational climate created.

## 5. Conclusions

It was shown that players’ immediate sports environment, created based on coach and peer motivational climate, is a critical factor in avoidance behaviours and fear of failure in handball practice.

The results obtained reinforce the importance of supportive behaviour and of promoting a coach and peer task climate, both during training and in competition and not just results. Likewise, athletes should be involved in task-oriented criteria for analysing sports failure and success, not only as a means to improve sports results, but also to make them value error as a means of learning, thus reducing their fear of failure and favouring satisfactory sports experiences and sports commitment. 

However, the results also suggest the need for parents, managers, and especially coaches to remain focused on the motivational climate in their team players, given that even when a coach adopts a positive attitude, the motivational climate in the team can negatively affect players’ sports experience and create a fear of failure.

## Figures and Tables

**Table 1:** Descriptive statistics, Cronbach’s alpha and correlations between the subscales of the Perceived Motivational Climate in Sport Questionnaire-2 (PMCSQ-2), the Performance Failure Appraisal Inventory (PFAI), and the Peer Motivational Climate in Youth Sport Questionnaire (PeerMCYSQ).

Subscales	M	SD	α	2	3	4	5	6	7	8	9
**PMCSQ-2**											
1. Coach ego climate	2.71	0.73	0.85	−0.39 **	0.24 **	0.27 **	0.35 **	0.42 **	0.36 **	0.49 **	0.47 **
2. Coach task climate	3.97	0.63	0.87		−0.14 **	−0.13 **	−0.16 **	−0.32 **	−0.21 **	0.46 **	−0.27 **
**PFAI** (Fear of …)											
3. Experiencing shame and embarrassment	2.62	0.95	0.85			0.69 **	0.53 **	0.56 **	0.54 **	0.05	0.25 *
4. Devaluing one’s self-estimate	2.48	0.85	0.70				0.63 **	0.53 *	0.56 **	0.10 *	0.23 **
5. Having an uncertain future	2.26	0.80	0.83					0.62 **	0.66 **	0.14 **	0.28 **
6. Important others losing interest	1.99	0.86	0.84						0.72 **	0.10 *	0.40 **
7. Upsetting important others	2.11	0.86	0.81							0.11 *	0.34 **
**PeerMCYSQ**											
8. Peer task climate	3.42	0.45	0.89								0.21 **
9. Peer ego climate	3.70	0.98	0.70								

Note: * *p* < 0.05; ** *p* < 0.01; M = mean; SD = standard deviation; α = Cronbach’s alpha.

**Table 2:** Multivariate analysis (intersubject effects according to gender and years of sports experience) based on the subscales of PMCSQ-2, PFAI, and PeerMCYSQ.

Subscales	Gender							Years of Sports Experience						
	Boys		Girls		F	*p*	Partial eta squared	≤5 years		>5 years		F	*p*	Partial eta squared
	M	ST	M	SD				M	SD	M	SD			
Coach ego climate	2.82	0.70	2.59	0.75	4.82	0.029	0.010	2.70	0.83	2.71	0.72	0.05	0.943	0.000
Coach task climate	3.87	0.66	4.08	0.57	2.06	0.152	0.004	4.01	0.59	3.97	0.63	0.18	0.671	0.000
Experiencing shame and embarrassment	2.53	0.94	2.73	0.94	3.75	0.053	0.008	2.34	0.94	2.67	0.94	8.02	0.005	0.017
Devaluing one’s self-estimate	2.46	0.89	2.50	0.81	0.55	0.460	0.001	2.27	0.80	2.52	0.86	5.38	0.021	0.011
Having an uncertain future	2.30	0.87	2.22	0.71	0.65	0.422	0.001	2.09	0.72	2.30	0.81	4.20	0.041	0.009
Important others losing interest	2.14	0.92	1.82	0.76	5.31	0.022	0.011	1.80	0.78	2.02	0.87	3.69	0.055	0.008
Upsetting important others	2.25	0.93	1.96	0.75	3.78	0.052	0.002	1.92	0.69	2.15	0.88	3.77	0.053	0.008
Peer task climate	3.44	0.44	2.59	0.76	0.84	0.359	0.002	3.46	0.45	3.41	0.45	0.75	0.387	0.002
Peer ego climate	4.03	0.87	3.33	0.96	3.49	0.000	0.068	3.47	0.96	3.74	0.98	3.63	0.057	0.008

Note: M = mean; SD = standard deviation.

**Table 3:** Linear regression analysis. Predictive subscales of the PMCSQ-2, the PeerMCYSQ, and the PFAI variable criterion.

Subscales	Fear of Experiencing Shame and Embarrassment					
	F	B	Beta	R^2^	t	*p*
Phase 1						
Peer ego climate	30.84	0.24	0.25	0.08	5.55	0.000
Phase 2						
Peer ego climate	20.63	0.17	0.17	0.10	3.47	0.001
Coach ego climate		0.20	0.16		3.14	0.002
	**Fear of Devaluing One’s Self-Esteem**					
	**F**	**B**	**Beta**	**R^2^**	**t**	***p***
Phase 1						
Coach ego climate	36.97	0.31	0.27	0.07	6.08	0.000
Phase 2						
Coach ego climate	22.06	0.24	0.21	0.08	4.18	0.000
Peer ego climate		0.11	0.13		2.59	0.010
	**Fear of Having an Uncertain Future**					
	**F**	**B**	**Beta**	**R^2^**	**t**	***p***
Phase 1						
Coach ego climate	64.73	0.38	0.35	0.12	8.05	0.000
Phase 2						
Coach ego climate	37.62	0.30	0.28	0.13	5.73	0.001
Peer ego climate		0.12	0.15		3.06	0.002
	**Fear of Important Others Losing Interest**					
	**F**	**B**	**Beta**	**R^2^**	**t**	***p***
Phase 1						
Coach ego climate	102.24	0.49	0.42	0.18	10.11	0.000
Phase 2						
Coach ego climate	71.33	0.35	0.30	0.23	6.51	0.000
Peer ego climate		0.23	0.26		5.78	0.000
Phase 3						
Coach ego climate		0.29	0.24		5.18	0.000
Peer ego climate	53.25	0.22	0.24	0.25	5.40	0.000
Coach task climate		–0.22	–0.16		–3.66	0.000
	**Fear of Upsetting Important Others**					
	**F**	**B**	**Beta**	**R^2^**	**t**	***p***
Phase 1						
Coach ego climate	69.40	0.42	0.36	0.13	8.33	0.000
Phase 2						
Coach ego climate	47.45	0.29	0.25	0.16	5.29	0.000
Peer ego climate		0.20	0.22		4.73	0.000

Note: R^2^ = Explained variance.

## References

[1] Atkins M.R., Johnson D.M., Force E.C., Petrie T.A. (2015). Peers, parents, and coaches, oh my! The relation of the motivational climate to boys’ intention to continue in sport. Psychol. Sport Exerc..

[2] Roberts G.C., Treasure D. (2012). Advances in Motivation in Sport and Exercise.

[3] Duda J.L., Ntoumanis N., Mahoney J.L., Larson R.W., Eccles J.S. (2005). After-School Sport for Children: Implications of a Task-Involving Motivational Climate.

[4] Moreno J.A., Conte L., Martínez C., Alonso N., González-Cutre D., Cervelló E. (2011). Psychometric properties of the Peer Motivational Climate in Youth Sport Questionnaire (PEERMCYSQ) with a sample of spanish athletes. Rev. Psicol. Deport..

[5] Ntoumanis N., Vazou S., Duda J.L., Jowette S., Lavallee D. (2007). Peer-created motivational climate. Social Psychology in Sport.

[6] Castillo I., Balaguer I., Duda J.L., García-Merita M.L. (2004). Psychosocial factors associated with sports practice during adolescence. Rev. Lat. Am. Psicol..

[7] Moreno-Murcia J.A., Conte L. (2011). Prediction of fear to err in basketball players through the peer motivational climate and intrinsic motivation. Rev. Mex. Psicol..

[8] Duda J.L., Balaguer I., Lavalee D., Jowett S. (2007). The coach-created motivational climate. Social Psychology of Sport.

[9] Granero-Gallegos A., Gómez-López M., Baena-Extremera A., Abraldes J.A., Rodríguez-Suárez N. (2012). Self-determined motivation in amateur handball. Rev. Iberoam. Diagn. Eval. Psicol..

[10] Gómez-López M., Granero-Gallegos A., Baena-Extremera A., Abraldes J.A. (2014). Goal orientation effects on elite handball players motivation and motivational climate. Procedia Soc. Behav. Sci..

[11] Ortiz P., Gómez-López M., Martín I., Reigal R.E., García-Mas A., Chirosa L.J. (2016). Role played by the coach in the adolescent players’commitment. Stud. Psychol..

[12] Granero-Gallegos A., Gómez-López M., Rodríguez-Suárez N., Abraldes J.A., Alesi M., Bianco A. (2017). Importance of the motivational climate in goal, enjoyment, and the causes of success in handball players. Front. Psychol..

[13] Moreno-Murcia J.A., Conte L., Silveira Y., Ruiz L. (2014). Fear of Failing in Sport.

[14] Ames C., Roberts G.C. (1992). Achievement goals, motivational climate, and motivational processes. Motivation in Sport and Exercise.

[15] Nicholls J.G. (1989). The Competitive Ethos and Democratic Education.

[16] Biddle S.J.H., Cury F., Goudas M., Sarrazin P., Famose J.P., Durand M. (1995). Development of scales to measure perceived physical education class climate: A cross-national project. Br. J. Educ. Psychol..

[17] Papaioannou A. (1994). Development of a questionnaire to measure achievement goals in physical education. Res. Q. Exerc. Sport..

[18] Kavussanu M., Roberts G.C. (1996). Motivation in physical activity contexts: The relationship of perceived motivational climate to intrinsic motivation and self-efficacy. J. Sport Exerc. Psychol..

[19] Newton M., Duda J.L., Yin Z. (2000). Examination of the psychometric properties of the Perceived Motivational Climate in Sport Questionnaire-2 in a simple of female athletes. J. Sports Sci..

[20] Seifriz J.J., Duda J.L., Chi L. (1992). The relationship of perceived motivational climate and intrinsic motivation and beliefs about success in basketball. J. Sport Exerc. Psychol..

[21] Moreno-Murcia J.A., Martínez-Galindo C., Belando N., Conte L. (2010). Relationship of the climate task of the equals with the fear to be wrong in the sport. Differences by sex in a sample of basketball players. Proceedings of the I International Congress of Culture and Gender: Culture in the Body.

[22] Nicholls J.G., Ames R., Ames C. (1984). Conceptions of ability and achievement motivation. Research on Motivation in Education: Student Motivation.

[23] Cecchini-Estrada J.A., de González-González Mesa C., Montero-Méndez J. (2008). Participation in sport, goal orientation and moral functioning. Rev. Lat. Am. Psicol..

[24] Sagar S.S., Lavallee D., Spray C.M. (2007). Why young elite athletes fear failure: Consequences of failure. J. Sport Sci..

[25] Ruiz-Sánchez V., Gómez-López M., Granero-Gallegos A. (2017). Motivational climate and fear of failure in the youthful teams of handball. Rev. Cienc. Deport..

[26] Ruiz-Sánchez V., Gómez-López M., Granero-Gallegos A., González-Hernández J. (2017). Relationship of motivational climate and fear of failure in high performance players in handball. Cuad. Psicol. Deport..

[27] Conroy D.E. (2001). Progress in the development of a multidimensional fear of failure measurement: The Performance Failure Appraisal Inventory (PFAI). Anxiety Stress Coping.

[28] Conroy D.E., Willow J.P., Metzler J.N. (2002). Multidimensional fear of failure measurement: The performance failure appraisal Inventory. J. Appl. Sport Psychol..

[29] Sagar S.S., Lavallee D., Spray C.M. (2009). Coping with the effects of fear of failure: A preliminary investigation of young elite athletes. J. Clin. Sport Psychol..

[30] Conroy D.E., Metzler J.N., Hofer S.M. (2003). Factorial invariance and latent mean stability of performance failure appraisals. Struct. Equ. Model..

[31] Sagar S.S., Jowett S. (2010). Validation of a multidimensional measure of fear of failure in a british sample: The performance failure appraisal inventory (PFAI). Int. J. Sports Sci. Coach..

[32] Tsai Y.M., Chen L.H. (2009). Relation of motivational climate and fear of Failure in taiwanese adolescent athletes. Psychol. Rep..

[33] Correia M., Rosado A., Serpa S. (2016). Fear of failure in sport: A portuguese crosscultural adaptation. Motriz Rev. Educ. Fís..

[34] Correia M., Rosado A., Serpa S. (2017). Psychometric properties of the Portuguese version of the Frost Multidimensional Perfectionism Scale. Int. J. Psychol. Res..

[35] Correia M., Rosado A., Serpa S., Ferreira V. (2017). Fear of failure in athletes: Gender, age and type of sport differences. Rev. Iberoam. Psicol. Ejerc. Deport..

[36] Wikman J.M., Stelter R., Melzer M., Hauge M.-L.T., Elbe A.-M. (2014). Effects of goal setting on fear of failure in young elite athletes. Int. J. Sport Exerc. Psychol..

[37] Kahraman N., Sungur S. (2016). Adaptation of the Performance Failure Appraisal Inventory (PFAI) into Turkish. Ahi Evran Üniversitesi Kırşehir Eğitim Fakültesi Dergisi.

[38] Alkhazaleh Z.M., Mahasneh A.M. (2016). Fear of failure among a sample of Jordanian undergraduate students. Psychol. Res. Behav. Manag..

[39] Moreno-Murcia J.A., Silveira Y., Conte L. (2013). Relatioship between positive feedback and the fear of failure of intrinsic motivation. Rev. Esp. Orientac. Psicopedag..

[40] Silveira Y., Moreno J.A. (2015). Fear of failure and self-determined motivation among adolescent students. Cuad. Psicol. Deport..

[41] Walling M.D., Duda J.L., Chi L. (1993). The perceived motivational climate in sport questionnaire: Construct and predictive validity. J. Sport Exerc. Psychol..

[42] Balaguer I., Guivernau M., Duda J.L., Crespo M. (1997). Analysis of the validity of the construct and the predictive validity of the questionnaire of motivational climate perceived in sport (PMCSQ-2) with Spanish competition tennis players. Rev. Psicol. Deport..

[43] Balaguer I., Mayo C., Atienza F.L., Duda J.L. (1997). Factorial validity of the Perceived Motivational climate in Sport Questionnaire-2 in the case of Spanish elite female handball teams. J. Sport Exerc. Psychol..

[44] Ntoumanis N., Vazou S. (2005). Peer motivational climate in youth sport: Measurement development and validation. J. Sport Exerc. Psychol..

[45] Bollen K., Long J.S. Testing Structural Equation Models.

[46] Tabachnick B.G., Fidell L.S. (2007). Using Multivariate Statistics.

[47] Hair J.F., Black W.C., Babin B.J., Anderson R.E. (2010). Multivariate Data Analysis.

[48] Gil J.A. (2003). Research Methods in Education. Multivariate Analysis.

[49] Horn T.S., Weiss M.R. (1991). A developmental analysis of children’s self-ability judgments in the physical domain. Pediatr. Exerc. Sci..

[50] Çağlar E., Aşçi F.H., Uygurtaş M. (2017). Roles of perceived motivational climates created by coach, peer, and parent on dispositional flow in young athletes. Percept. Motor Skills.

[51] Iwasaki S., Fry M.D. (2016). Female adolescent soccer players’ perceived motivational climate, goal orientations, and mindful engagement. Psychol. Sport Exerc..

[52] Jaakkola T., Ntoumanis N., Liukkonen J. (2016). Motivational climate, goal orientation, perceived sport ability, and enjoyment within Finnish junior ice hockey players. Scand. J. Med Sci. Sport.

[53] Marques M., Nonohay R., Koller S., Gauer G., Cruz J. (2015). Coaches’ communication style and the perception of the motivational climate created by coaches and teammates. Cuad. Psicol. Deport..

[54] Møllerløkken N., Lorås H., Pedersen A. (2017). A comparison of players’ and coaches’ perceptions of the coach-created motivational climate within youth soccer teams. Front Psychol..

[55] Torregrosa M., Viladrich C., Ramis Y., Azócar F., Latinjak A.T., Cruz J. (2011). Effects on the perception of the motivational climate created by coaches and teammates on enjoyment and commitment. Gender differences. Rev. Psicol. Deport..

[56] Barkoukis V., Koidou E., Tsorbatzoudis H. (2010). Effects of a motivational climate intervention on state anxiety, self-efficacy, and skill development in physical education. Eur. J. Sport Sci..

[57] Smith R.E., Smoll F.L., Cumming S.P. (2007). Effects of a motivational climate intervention for coaches on Young Athletes’ sport performance anxiety. J. Sport Exerc. Psychol..

[58] Abraldes J.A., Granero-Gallegos A., Baena-Extremera A., Gómez-López M., Rodríguez-Suárez N. (2016). Goal orientations, satisfaction, beliefs in sport success and motivational climate in swimmers. Rev. Int. Med. Cienc. Act..

[59] Leo F.M., Sánchez-Miguel P.A., Sánchez-Oliva D., Amado D., García-Calvo T. (2015). Motivational climate created by other significant actors and antisocial behaviors in youth sport. Kinesiology..

[60] Conroy D.E. (2004). The unique psychological meanings of multidimensional fears of failing. J. Sport Exerc. Psychol..

[61] Sagar S.S., Busch B., Jowett S. (2010). Success and failure, fear of failure, and coping responses of adolescent Academy Football Players. J. Appl. Psychol..

[62] Sagar S.S., Stoeber J. (2009). Perfectionism, fear of failure and affective responses to success and failure: The central role of fear of experiencing shame and embarrassment. J. Sport Exerc. Psychol..

[63] Gustafsson H., Sagar S.S., Stenling A. (2017). Fear of failure, psychological stress, and burnout among adolescent athletes competing in high level sport. Scand. J. Med. Sci. Sport.

[64] Boixadós M., Cruz J., Torregrosa M., Valiente L. (2004). Relationships among motivational climate, satisfaction, perceived ability, and fair play attitudes in young soccer players. J. Appl. Sport Psychol..

[65] Smith R., Cumming S., Smoll F. (2008). Development and validation of the motivational climate scale for youth sports. J. Appl. Sport Psychol..

[66] Gábor G., Géza V., Miklós K., József B. (2009). Elite young team players’ coping, motivation and perceived climate measures. Phys. Cult. Sport Stud. Res..

[67] Moreno J.A., Cervelló E., González-Cutre D. (2007). Young athletes’ motivational profiles. J. Sports Sci. Med..

[68] Moreno J.A., Cervelló E., González-Cutre D. (2008). Relationships among goal orientations, motivational climate and flow in adolescent athletes: Differences by gender. Span. J. Psychol..

[69] Vazou S., Ntoumanis N., Duda J.L. (2006). Predicting young athletes’ motivational indices as a function of their perceptions of the coach- and peer-created climate. Psychol. Sport Exerc..

[70] White S.A., Duda J.L. (1994). The relationship of gender, level of sport involvement, and participation motivation to task and ego orientation. Int. J. Sport Psychol..

[71] White S.A., Kavussanu M., Guest S.M. (1998). Goal orientations and perceptions of the motivational climate created by significant others. Eur. J. Phys. Educ.

[72] Galván J.F., López-Walle J., Pérez J.A., Tristán J.L., Medina R.E. (2013). Motivational climate in individual sports and set in mexican youth athletes. Rev. Iberoam. Psicol. Ejerc. Deport..

[73] McGregor H.A., Elliot A.J. (2005). The same of failure: Examining the link between fear of failure and shame. Pers. Soc. Psychol. B.

[74] Moreno-Murcia J.A., Ferriz R., Belando N., Conte L. (2011). Fear of failure in young basketball players: Differences by sex. Proceedings of the II Congress of School-Age Sports.

[75] Conroy D.E., Coatsworth J.D., Kaye M.P. (2007). Consistency of fear of failure score meanings among 8- to 18-year-old female athletes. Educ. Psychol. Meas..

[76] Ruiz-Sánchez V., Gómez-López M., Herrera Cuadrado J.L. (2017). Observational analysis of handball shot in the counterattack phase of the national teams finalists in 2015 Qatar world handball cup. Espiral.

